# Socioeconomic inequality in informal payments for health services among Iranian households: a national pooled study

**DOI:** 10.1186/s12889-023-15071-6

**Published:** 2023-02-23

**Authors:** Jafar Yahyavi Dizaj, Maryam Khoramrooz, Vajihe Ramezani-Doroh, Satar Rezaei, Reza Hashempour, Kamran Irandoust, Shahin Soltani, Ali Kazemi-Karyani

**Affiliations:** 1 Research Center for Environmental Determinants of Health, Health Institute, Kermanshah University of Medical Sciences, Kermanshah, Iran; 2 Department of Health Management and Economics, School of Public Health, Hamadan University of Medical Sciences, Hamadan, Iran; 3 Modeling of Noncommunicable Diseases Research Center, Hamadan University of Medical Sciences, Hamadan, Iran; 4 Office in Treatment Affairs, Esfarayen Faculty of Medical Sciences, Esfarayen, Iran; 5 Department of Health Economics, School of Health Management and Information Sciences, Iran University of Medical Sciences, Tehran, Iran

**Keywords:** Socioeconomic inequality, Informal payments, Health system, Households, Iran

## Abstract

**Background:**

There is limited evidence on the prevalence and socioeconomic inequality in informal payments (IP) of households in the Iranian health system. This study was conducted to investigate the prevalence of IP and related socioeconomic inequalities among Iranian households in all provinces.

**Method:**

Data on Household Income and Expenditure Surveys (HIES) for 91,360 households were used to examine the prevalence and inequality in informal health sector payments in the years 2016 to 2018. The Normalized Concentration Index (NC) was used to examine inequality in these payments and the decomposition analysis by the Wagstaff approach was used to determine the share of variables affecting the measured inequality.

**Results:**

Of the total households, 7,339 (7.9%) reported IP for using health services. Urban households had higher IP (10%) compared to rural ones (5.42%). Also, the proportion of households with IP in 2016 (11.69%) was higher than in 2017 (9.9%), and 2018 (4.60%). NC for the study population was 0.129, which shows that the prevalence of IP is significantly higher in well-off households. Also, NC was 0.213 (*p* < 0.0001) and -0.019 for urban and rural areas, respectively (*p* > 0.05). Decomposition analysis indicated that income, sex of head of household, and the province of residence have the highest positive contribution to measured inequality (with contributions of 156.2, 45.8, and 25.6%, respectively).

**Conclusion:**

There are a significant prevalence and inequality in IP in Iran's health system and important variables have shaped it. On the whole, inequality was pro-rich. This may lead to increasing inequality in access to quality services in the country. Our findings showed that previous health policies such as regulatory tools, and the health transformation plan (HTP) have not been able to control IP in the health sector in the desired way. It seems that consumer-side policies focusing on affluent households, and high-risk provinces can play an important role in controlling this phenomenon.

**Supplementary Information:**

The online version contains supplementary material available at 10.1186/s12889-023-15071-6.

## Introduction

Health has always been one of the most important concerns and basic human needs [1] and the government is not only responsible for meeting these needs [2] but also is responsible for the financial protection of individuals and households from health expenditures [3]. Taxes, insurance, and out-of-pocket payments are the most common methods of financing health care services [4], each of which has different effects on health system indicators, and some of its forms, such as out-of-pocket payments, may result in corruption [5].

Low payments, lack of sufficient resources to meet needs in environments with poor governance, low transparency, and poor response mechanisms are among the causes of corruption [6]. The health sector is attractive to corruption for various reasons such as lack of patient information, symmetry of information between the patient and the service provider, and the economic benefits of providing service to the patient [7].

One of the types of corruption in the health system is IP, which is very common [8, 9]. IP generally mean any payment to individual providers and institutions, in the form of goods or cash, apart from official payment channels or purchases covered by the health care system [10]. These payments are often made in order to improve access to health services, avoid queuing to receive high-quality services, or give thanks to the provider(s) [11].

Studies have shown that one of the shortcomings of the Iranian health system is IP [4, 12, 13]. To overcome this shortcoming, the government implemented a series of reforms under the Health Transformation Plan (HTP) in 2014, one of the most important goals of which was to reduce IP. However, according to the results of the study by Doshmangir et al. (2020), although IP has decreased, it is still common in Iran's health system [14].

In Iran, some studies have examined IP in the health sector. In a study conducted by Khodamoradi et al. (2013) in the hospitals of Urmia, it was found that twelve percent of the discharged patients had paid doctors an average of 5,030,000 Iranian Rials ($ 412). About 5% of patients paid IP to nurses and 17% to other staff. Hospital ownership, patient income, place of residency, literacy, and surgery performed have been introduced as factors affecting IP [15].

The study of Mesgarpour et al. (2018) also showed that 27.7% of the 1112 samples in this study had at least one experience of IP. The prevalence of voluntary and mandatory IP is 11.5 and 21.5%, respectively, and the prevalence of IP is higher in hospitalized patients than in outpatients [16]. Another study in hospitals in Kurdistan province showed that the percentage of IP to doctors in hospitals affiliated to the Ministry of Health, Social Security and the private sector were 4.5%, 8.1%, and 12.5%, respectively before implementation of HTP. However, after the first phase of the implementation of HTP those figures were 0%, 7.1%, and 10%, respectively [17].

IP is higher among younger people, women, married people, employed people, low-income groups, and rural people. Also, socioeconomic, moral, legal and governance failures are determinants of IP [18]. Another important issue in financing the health system is the existence of socioeconomic inequalities [19]. A survey of 33 African countries over several periods has shown that these payments are focused on poor people and that the failure to provide health services has contributed the most to this inequality [11].

The available evidence on the prevalence of socioeconomic inequality in IP of the Iranian health system is very limited. Therefore, this study was conducted to investigate the prevalence of informal health payments and related socioeconomic inequalities among Iranian households in different provinces to provide new evidence of the status of these payments to policymakers and researchers in the health system.

## Methods

### Study area

Iran is a developing country with a health system that compromises with a mix of public and private sectors. The public health centers provide three levels of preventive, curative, and rehabilitation services for people. Most preventive services such as vaccination, maternal care, elderly care, etc. are provided for free [20]. Since the implementation of HTP in 2014 cost-sharing for insured people decreased for most healthcare services such as inpatient ones. Private health care mostly provides curative and rehabilitation services for people that can afford them. The private health sector has a higher relative price than the public sector and usually is concentrated in big and affluent cities. Financing for the health system also is a mix of public and private resources such as government budget, taxes, premiums, and out-of-pocket payments (OOP) [21]. Social Security Organization and Iranian Health Insurance Organization have the highest share of health insurance for the population and private insurance companies often provide complementary health insurance [22]. Some evidence shows that in most years more than 50% of health expenditures come from OOP. Therefore, one of the main goals of national development programs has been to decrease OOP and catastrophic health expenditures [23]. Also, IP is one of the problems of the health system, so it has been reported by about 27% of those who used health services. Therefore, decreasing OOP and IP in the health system has been targeted in HTP. Also, catastrophic health expenditures have a high prevalence in this health system [24]. This study aimed to investigate the prevalence of IP and related socioeconomic inequality among Iranian households after the implementation of HTP.

### Data and variables

In this study, data on the Household Income and Expenditure Survey (HIES) of the Iran Statistical Center (ISC) from 2016 to 2018 were used for analysis. HIES is a large cross-sectional study conducted annually by Iran's statistics center at the level of households in the entire country and this data is representative of Iranian households. The data of HIES collect using a three-stage random cluster sampling method and covers all the provinces including urban and rural areas of the country. Three phases of sampling were as follows: 1. Classification of study areas (urban and rural areas); 2. Random selection of the study blocks; 3. Random selection of households in selected blocks. In the second phase, the number of blocks in urban or rural areas of each province has been determined proportional to the population of each area, and in the third phase, households were selected randomly in urban and rural blocks. The weighting of households was done based on the probability of choosing the sample household from the total number of households in each block. So, the households' weight in each block was calculated as the inverse of the sampling probability (the ratio of the population to the number of samples chose from the block). All estimates, including percentages, concentration index, and decomposition analysis, have been made by taking into account the sampling weights.

Households that do not respond replace with another household as an alternative sample. The total number of households included in the three years of this study was 115,070 (38,146, 37,962, and 38,962 households in 2016, 2017, and 2018, respectively). Of these, 91,360 households had used healthcare services and paid for health services. Of this number, 91,204 household data were included in the final analysis after data refining.

The outcome variable is having IP for health services. If a household had paid for health services in any way outside of the bill of healthcare centers during the past year, it was considered as having IP. Using the obtained data, ISC divides the households according to their income into 10 groups. Therefore, to determine the economic status of households, we divided households into five groups based on income deciles of households. Income compromises all earnings of households including salaries, wages, earnings from properties, rent, cash subsidies, etc. during the month before the survey by ISC. The income variable was used as a ranking variable in measuring economic inequality. Other dependent variables include sex of head of household, age of head of household, education level of head of household, employment of head of household, place of residence, having < 12 years old member, having > 65 years old member, having a member with a special disease in a household, health insurance coverage, and year of the survey were extracted from available data.

### Inequality measurement

Socioeconomic-related inequality was measured by Wagstaff normalized concentration index (NC). The following formula was used to measure NC:

$$NC= \frac{2}{n\mu (1-\mu )} \sum_{i=1}^{n}{y}_{i}{r}_{i}-1$$

where here,${y}_{i}$ is the facing with IP in the health system for $i$-th household and ${r}_{i}$ is the fractional rank of $i$-th household in the distribution of household's income and $\mu$ is the mean of the outcome variable (facing IP). The NC value varies between -1 and + 1 so that the zero shows that the distribution of the outcome variable is equal among households with different SES ranks. The positive (negative) values of NC show that there is inequality in favor of the poor (the rich) households.

### Decomposition of inequality

Wagstaff decomposition approach was used to obtain the contribution of explanatory variables such as sex and age of head of households, place of residence, etc. to the measured inequality in the outcome variable (IP). Consequently, considering a linear relation between IP and its determinants, the NC rewrites as follows:

$$NC={\sum }_{k}(\frac{{\beta }_{k}{\overline{X} }_{k}}{\mu }){C}_{k}+\frac{{C}_{e}}{\mu }={\sum }_{k}{\eta }_{k}{C}_{k}+\frac{{C}_{e}}{\mu }={C}_{\widehat{y}}+\frac{{C}_{e}}{\mu }$$

where here:

$NC$ was decomposed to the attributable inequality to variations of the explanatory variables among different income quintiles (${C}_{\widehat{y}}$) and residual part ($\frac{{C}_{e}}{\mu }$) which is an unexplained part of the measured inequality. ${\beta }_{k}$ is marginal effects of the explanatory variables that were obtained by performing a logistic regression model. Furthermore, $\left(\frac{{\beta }_{k}{\overline{X} }_{k}}{\upmu }\right)$ signifies the elasticity of explanatory variables, where here ${\overline{X} }_{k}$ is the proportion (mean) of explanatory variable k (x_k_). In this equation ($\upmu$) is the mean of the outcome variable of facing informal payment.

A positive (negative) contribution of an explanatory variable to the measured inequality (NC) shows that the relationship between this variable and facing informal payment (outcome variable) contributes to a lower likelihood of outcome variable among the poor (the rich). In the last steps of decomposition analysis, by multiplying the elasticity of each explanatory variable by its concentration index (${\eta }_{k}{C}_{k}$), the absolute contribution of each explanatory variable to the total inequality was measured. Also, the percentage contribution of each explanatory variable was calculated by dividing the absolute contribution of that variable to NC of the distribution of informal payment among households. The software of Access and Excel version 2013 was used for data extraction and all other analyses were performed by Stata version 14.2 (StataCorp, College Station, TX, USA).

## Results

A total of 91,204 households were included in the analysis, of which 7.9% had IP. IP was more prevalent among male-headed households. Households with over 65 years old, illiterate, and unemployed heads had the highest percentage of IP compared to their counterparts. The prevalence of IP increased from 3.9% in 1^st^ income quintile to 11% in 5^th^ income quintile. Households with < 12 years old children had less percentage of IP than the other households. Furthermore, households with an > 65 years old member had more percentage of IP. The prevalence of IP among households with a member with a special disease was more than in other households, and IP was less prevalent among households with no health insurance coverage. Also, IP decreased from 11.6% in 2016 to 4% in 2018 (Table 1).

**Table 1:** Prevalence of informal payments for health services among Iranian households, 2016–18

Variables		Total (%)	Informal payment	
			Yes (%)	No (%)
Total		91,204 (100)	7339 (7.9)	83,865 (92.1)
Sex of head of household	Male	79,020 (86.96)	6545 (8.12)	72,475 (91.88)
	Female	12,184(13.04)	794(6,57)	11,390(93.43)
Age of head of household (years)	≤ 35	16,037(17.07)	1173(7.25)	14,864(92.75)
	36–50	31,269(34.7)	2297(7.29)	28,972(92.71)
	51–65	25,807(29.45)	2124(7.98)	23,683(92.02)
	> 65	18,091(18.78)	1745(9.58)	16,346(90.42)
Education level of head of household	Illiterate	23,574(19.18)	2080(8.63)	21,494(91.37)
	Non-academic	54,783(62.88)	4201(7.74)	50,582(92.26)
	Academic	12,847(17.94)	1058(7.8)	11,789(92.2)
Employment of head of household	Employed	60,885(64.12)	4721(7.6)	56,164(92.4)
	Unemployed	30,319(35.88)	2618(8.48)	27,701(91.52)
Place of residence	Rural	45,267(45.42)	3398(5.42)	42,539(94.58)
	Urban	45,937(54.58)	3941(10)	41,326(90)
Income quintiles	1^st^	15,996(13.91)	717(3.94)	15,279(96.06)
	2^nd^	17,695(17.31)	1197(6.35)	16,498(93.65)
	3^rd^	18,524(19.42)	1436(6.93)	17,088(93.07)
	4^th^	19,047(21.27)	1761(8.65)	17,286(91.35)
	5^th^	19,942(28.09)	2228(10.98)	17,714(89.02)
Having < 12 years old member in household	Yes	40,657(42.35)	3198(7.7)	37,459(92.3)
	No	50,547(57.65)	4141(8.07)	46,406(91.93)
Having > 65 years old member in household	Yes	22,285(23.10)	2198(9.87)	20,087(90.13)
	No	68,919(76.9)	5141(7.33)	63,778(92.67)
Having a member with the special disease in household	Yes	1378(1.75)	208(15.48)	1179(84.52)
	No	89,817(98.25)	7131(7.78)	82,686(92.22)
Health insurance coverage	Yes	74,064(75.3)	5778(7.5)	68,286(92.5)
	No	17,140(24.7)	1561(9.1)	15,579(90.9)
Year of survey	2016	26,271(28.69)	3109(11.62)	23,162(88.38)
	2017	26,083(29.41)	2444(9.9)	23,639(90.1)
	2018	38,850(41.90)	1786(4)	37,064(64)

^†^ Percentages were adjusted for sample weights

**Fig. 1:**
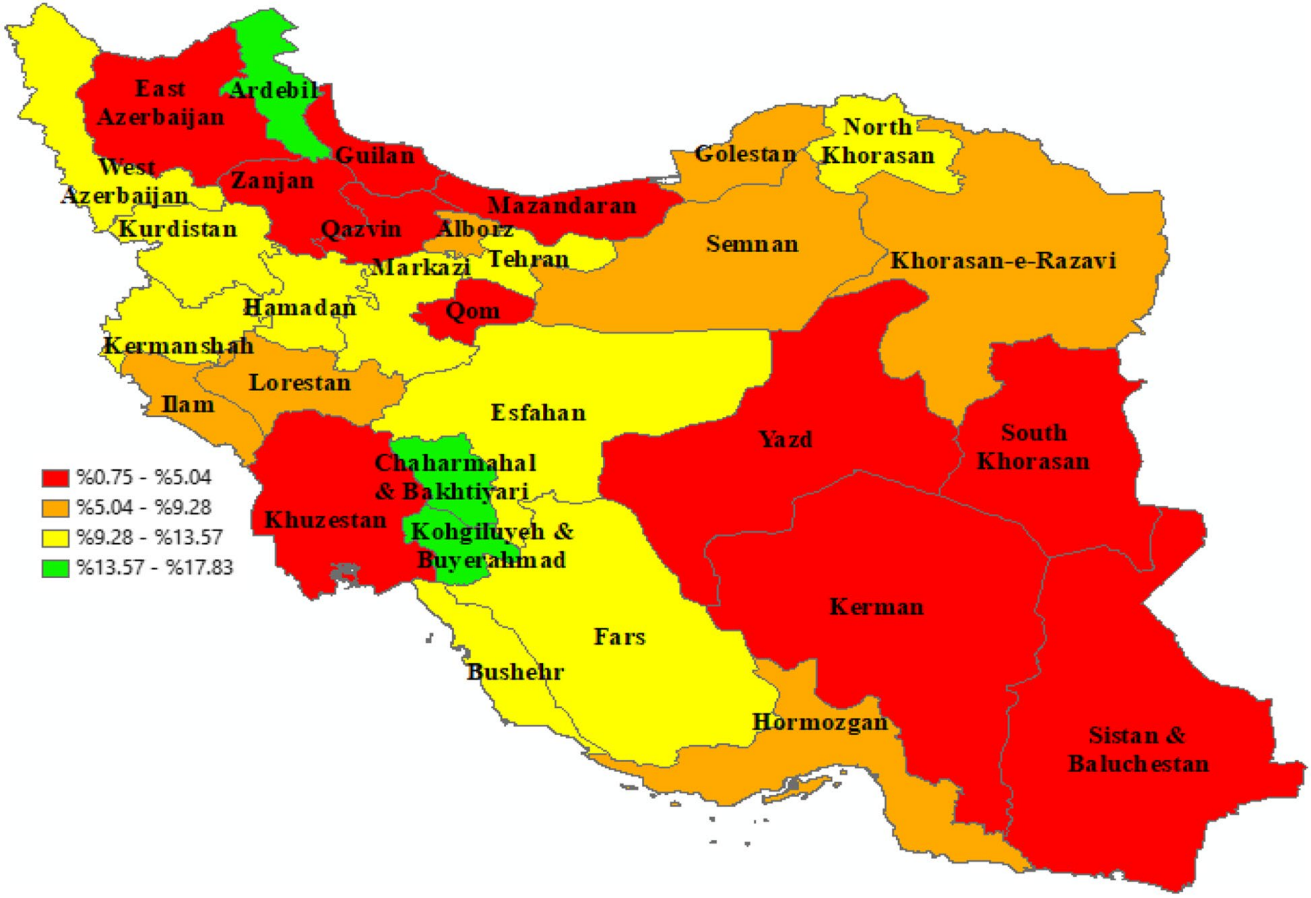
The prevalence of informal payments among Iranian households by province, 2016–18

Table 2 indicates the geographical distribution of prevalence and socioeconomic inequality of informal payments for health services among Iranian households. According to the results, IP was more prevalent among urban than rural households (10% vs. 5.4%). The highest prevalence of IP was observed in Ardabil (17.8%), Chaharmahal and Bakhtiari (16.9%), and Kohgoluyeh and Boyer-Ahmad (15.3%) provinces, whereas, Khuzestan (1.7%), Guilan (1.1%), and Sistan-Baluchestan (0.8%) provinces had the lowest percentages of IP in the country. Figure 1 depicts the prevalence of IP among Iranian households by the province of residency.

**Table 2:** Geographical distribution of prevalence and socioeconomic inequality of informal payments for health services among Iranian households, 2016–2018

Province	Total (%)	Informal payment		NC
		**Yes (%)**	**No (%)**	
**Total**	91,204 (100)	7339 (7.92)	83,865 (92.08)	0.129^*^
**Rural areas**	45,937(54.58)	3941(10.00)	41,326(90.00)	-0.019
**Urban areas**	45,267(45.42)	3398(5.42)	42,539(94.58)	0.213^*^
**Provinces**				
Markazi	3502(1.92)	375(10.10)	3127(89.9)	0.070
Guilan	3070(3.62)	29(1.06)	3041(98.94)	0.057
Mazandaran	2742(4.66)	107(3.68)	2635(96.32)	0.149
Azerbaijan, East	3448(5.65)	185(4.89)	3263(95.11)	0.254
Azerbaijan, West	2425(3.39)	282(13)	2143(87)	0.165
Kermanshah	3324(2.59)	432(12.51)	2892(87.49)	0.051
Khuzestan	3155(5.33)	80(1.68)	3075(98.32)	-0.178
Fars	3579(6.02)	345(9.87)	3234(90.13)	0.106^*^
Kerman	2442(3.54)	130(4.95)	2312(95.05)	-0.021
Khorasan, Razavi	3837(7.87)	318(7.31)	3519(92.69)	0.022
Isfahan	3574(7.43)	401(9.61)	3173(90.39)	0.049
Sistan and Baluchestan	2402(2.10)	19 (0.75)	2383(99.25)	0.260
Kurdistan	1643(1.68)	164(11.14)	1479(88.86)	0.131
Hamadan	2814(2.12)	292(11.13)	2522(88.87)	0.138
Chaharmahal and Bakhtiari	2378(1.1)	411(16.90)	1967(83.1)	0.014
Lorestan	2484(2.07)	225(8.29)	2259(91.71)	0.099
Ilam	2506(0.68)	124(5.32)	2382(94.68)	0.130
Kohgiluyeh and Boyer-Ahmad	2738(0.78)	416(15.27)	2322(84.73)	-0.035
Bushehr	2444(1.22)	269(10.10)	2175(89.90)	0.004*10^-1^
Zanjan	2588(1.28)	57(2.03)	2531(97.97)	-0.088
Semnan	2336(.88)	139(6.51)	2197(93.49)	0.169
Yazd	2918(1.32)	85(1.69)	2833(98.31)	0.005
Hormozgan	3457(2.04)	327(8.71)	3130(91.29)	-0.027
Tehran	4658(17.17)	468(10.74)	4190(89.26)	0.171
Ardabil	2723(1.73)	466(17.83)	2257(82.27)	0.094
Qom	2604(1.78)	129(2.80)	2475(98.20)	-0.001
Qazvin	2537(1.76)	2461(2.30)	2461(97.97)	0.070
Golestan	3875(2.52)	297(8.55)	3578(91.45)	0.134
Khorasan, North	3226(1.05)	340(11.07)	2886(88.93)	0.125
Khorasan, South	3206(0.9)	110(3.11)	3096(96.89)	-0.127
Alborz	2569(3.75)	241(8.80)	2328(91.20)	0.237

^†^ Percentages were adjusted for sample weights

^*^p < 0.05

**Fig. 2:**
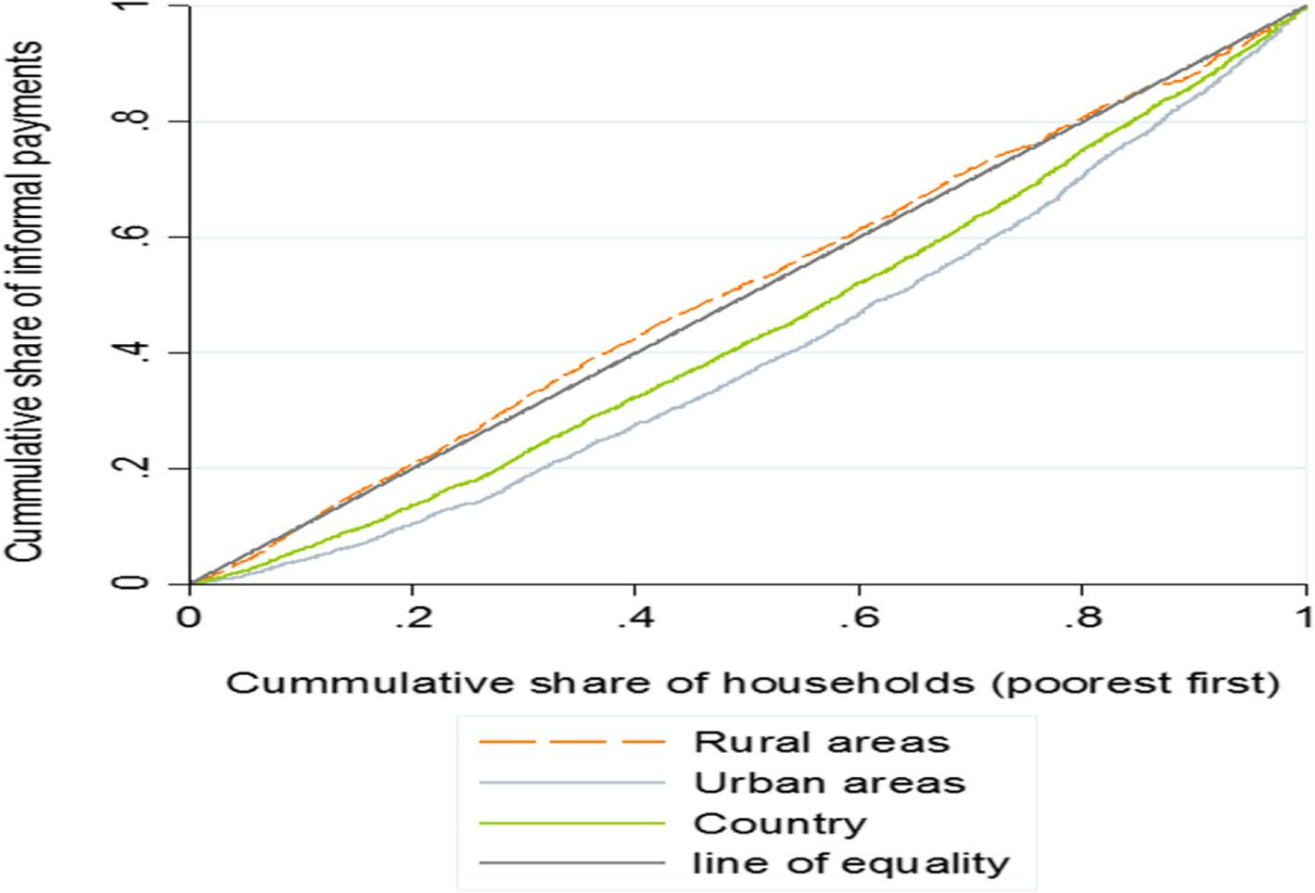
Concentration curves of informal payments for health services among Iranian households, 2016–2018

The NC of IP in the country was equal to 0.129, which shows that it was more prevalent among higher-income households. Also, the values ​​of concentration indices in urban and rural areas were 0.213 (*p* < 0.0001) and -0.019 (*p* > 0.05), respectively, which shows that while IP was more concentrated among higher-income households in urban areas, it was almost equally distributed among rural households from different income quintiles. As it is shown in Fig. 2, the concentration curve of IP in the country and urban areas were below the line of inequality, indicating its more concentration among high-income households. However, the concentration curve of IP in rural areas almost coincides with the line of inequality, which suggests its equal concentration among rural households from different income quintiles.

**Fig. 3:**
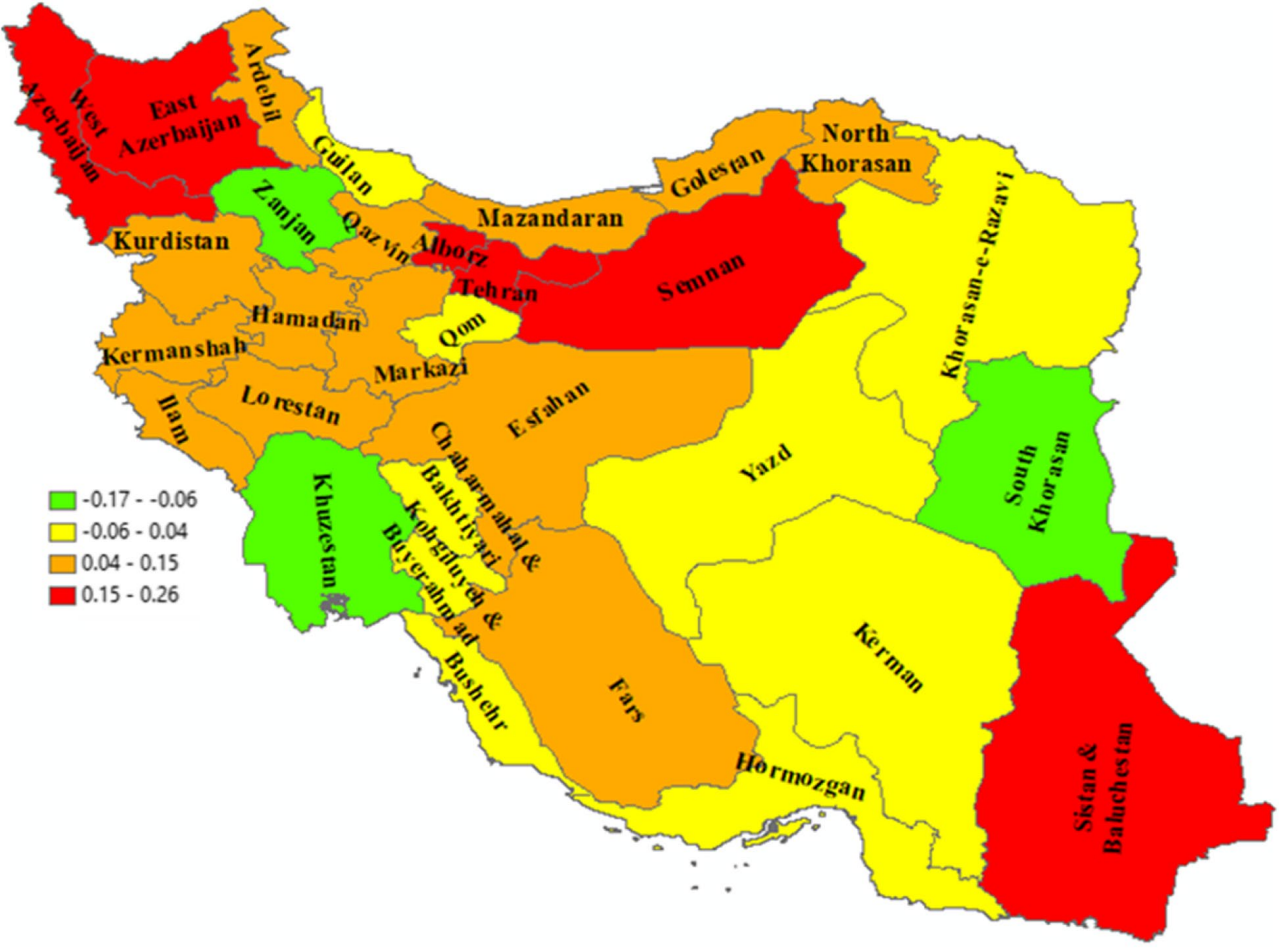
The normalized concentration index of informal payments among Iranian households by province, 2016–18

Also, the socioeconomic inequality in IP was significantly different among the provinces of the country (*p* < 0.0001). NC for East Azerbaijan, Alborz, Tehran, Semnan, West Azerbaijan, Mazandaran, Hamedan, Golestan, Kurdistan, Ilam, North Khorasan, Fars, Lorestan, Ardabil, and Markazi provinces was 0.254. 0.237, 0.171, 0.169, 0.165, 0.149, 0.138, 0.134, 0.131, 0.130, 0.106, 0.125, 0.099, 0.094, and 0.07, respectively. This findings showed more concentration of IP among high-income households in these provinces. In the Khuzestan and South Khorasan provinces with an NC of -0.178 and -0.127, respectively, IP was more concentrated among low-income households. In other provinces of the country, IP is almost equally distributed among households from different income quintiles (Table 2 and Fig. 3).

According to the findings income quintiles, gender and the province of residence have the highest positive contributions to measured inequality (with contributions of 156.1%, 45.8%, and 23.9%, respectively) (Table 3). Also, the difference in the research year, literacy of the head of household, and having a member over 65 years in the household were the most negative contributors (-0.31.2%, -29.1%, and -12.2%, respectively) to the inequality in IP.

**Table 3:** Decomposition analysis of socioeconomic inequality in informal payments among Iranian households, 2016–18

Variables	Coefficient	ME	Elasticity	CI (C_k_)	AC	PC	SPC
**Sex (RC: Female)**							
Male	0.233	0.016	0.176	0.334	0.059	45.8	45.8
**Age of Head of Household (RC: > 65 years)**							
≤ 35	0.243	0.017	0.036	-0.134	-0.005	-3.8	1.7
36–50	0.091	0.006	0.028	0.115	0.003	2.5	
51–65	0.131	0.009	0.034	0.115	0.004	3	
**Education of head of household (RC: Academic)**							
Illiterate	0.498	0.034	0.083	-0.505	-0.042	-32.7	-29.1
Non academic	0.204	0.014	0.111	0.041	0.005	3.5	
**Employment of head of household (RC: Unemployed)**							
Employed	-0.107	-0.007	-0.060	0.120	-0.007	-5.6	-5.6
**Place of residence (RC: Rural)**							
Urban	0.233	0.016	0.111	-0.033	-0.004	-2.8	-2.8
**Income quintiles (RC: 5**^**th**^**)**							
1^st^	-1.192	-0.082	-0.144	-0.919	0.133	103.1	156.1
2^nd^	-0.674	-0.046	-0.102	-0.602	0.061	47.5	
3^rd^	-0.544	-0.037	-0.092	-0.215	0.020	15.4	
4^th^	-0.282	-0.019	-0.052	0.243	-0.013	-9.8	
**Having an < 12 years old member in household (RC: No)**							
Yes	0.125	0.009	0.046	0.019	0.001	0.7	0.7
**Having an > 65 years old member in household (RC: No)**							
Yes	0.444	0.031	0.089	-0.176	-0.016	-12.2	-12.2
**Having a member with special disease in household (RC: No)**							
Yes	0.562	0.039	0.009	0.188	0.002	1.3	1.3
**Health insurance coverage (RC: No)**							
Yes	0.052	0.004	0.034	0.041	0.001	1.1	1.1
**Year of Survey (RC: 2018(**							
2016	1.011	0.070	0.252	-0.165	-0.042	-32.4	-31.2
2017	0.831	0.057	0.213	0.008	0.002	1.3	
**Province (RC: Alborz)**							
Markazi	0.336	0.023	0.006	-0.128	-0.001	-0.6	23.9
Guilan	-2.084	-0.144	-0.066	-0.125	0.008	6.4	
Mazandaran	-0.916	-0.063	-0.037	0.096	-0.004	-2.8	
Azerbaijan, East	-.558	-0.038	-0.027	-0.108	0.003	2.3	
Azerbaijan, West	0.719	0.050	0.021	-0.257	-0.005	-4.2	
Kermanshah	0.491	0.034	0.011	-0.063	-0.001	-0.5	
Khuzestan	-1.699	-0.117	-0.079	0.026	-0.002	-1.6	
Fars	0.181	0.012	0.009	0.029	0.0003	.02	
Kerman	-0.196	-0.013	-0.006	-0.465	0.003	2.2	
Khorasan, Razavi	-0.027	-0.002	-0.002	-0.096	0.0002	0.1	
Isfahan	0.158	0.011	0.010	0.057	0.001	0.5	
Sistan and Baluchestan	-2.123	-0.147	-0.039	-0.420	0.016	12.7	
Kurdistan	0.500	0.034	0.007	-0.119	-0.001	-0.7	
Hamadan	0.419	0.029	0.008	-0.156	-0.001	-0.9	
Chahar Mahaal and Bakhtiari	0.841	0.058	0.008	0.012	0.0001	0.1	
Lorestan	0.133	0.009	0.002	-0.193	-0.0005	-0.4	
Ilam	-0.377	-0.026	-0.002	-0.207	0.0004	0.4	
Kohgiluyeh and Boyerahmad	0.796	0.055	0.005	-0.092	-0.0004	-0.4	
Bushehr	0.299	0.021	0.003	0.040	0.0001	0.1	
Zanjan	-1.380	-0.095	-0.015	-0.120	0.002	1.4	
Semnan	-0.032	-002	-0.000	-0.272	0.0001	0.1	
Yazd	-1.581	-0.109	-0.018	0.018	-0.0003	-0.3	
Hormozgan	0.103	0.007	0.002	-0.048	-0.00001	-0.1	
Tehran	0.194	0.013	0.029	0.426	0.014	10.5	
Ardabil	0.990	0.68	0.029	-0.155	-0.002	-1.8	
Qom	-0.536	-0.37	-0.008	-0.012	0.0001	0.1	
Qazvin	-1.455	-0.1	-0.022	-0.048	0.001	0.8	
Golestan	0.175	-0.012	-.004	-0.244	-0.001	-0.7	
Khorasan, North	0.503	0.035	0.005	-0.237	-0.001	-0.8	
Khorasan, South	-0.729	0.050	0.006	-0.427	0.002	1.9	
**Explained**					**0.193**		**149.6**
**Residual**					**-0.064**		**-49.6**
**Total**					**0.129**		**100**

**Abbreviations: *RC* Reference Category, *ME* Marginal effect, *CI* Concentration Index (C**_**k**_**), *AC* Absolute contribution, *SPC* Summed percentage contribution**

Considering the year of study, NC was 0.177 ± 0.011, 0.161 ± 0.012, and 0.165 ± 0.015 for 2016, 2017, and 2018, respectively. Decomposition analysis by years showed that income of households had the most contribution to inequality in IP for all years. In 2016, sex had the most positive contribution (53.2%) after income. On contrary, education of head of household had the most negative contribution in inequality (-18.7%) in 2016, and 2017. But place of residency (rural/urban) had the highest negative contribution in 2018 (-33.9%). Contribution of province increased from 1.5% in 2016 to 38.5% in 2018. Additional file 1 shows the decomposition analysis by years of study.

## Discussion

The present study ended to determine inequality in the IP and decompose the observed inequality into its determinants in Iran. The results showed that although the prevalence of these payments was different across provinces, in general, the prevalence of IP in Iran, compared to some studies [12, 18, 25–29], was relatively less, and was relatively more in comparison to others [3, 30, 31]. The prevalence of IP in the study period was 7.9% which ranged from 11.62% in 2016 to 4% in 2018. Another study reported a prevalence of IP 27.1% (2017) that 21.5% of them had been paid by force of providers. Also, this study indicated that some consumers paid for IP more than once [32]. Also, the study by Piroozi et al. (2017) in the west of Iran reported that the prevalence of receiving IP among physicians was 4.5%, and 12.5% in public and private hospitals, respectively. Meanwhile, this study indicated that IP decreased by 0 and 10 percent after the implementation HTP in public and private centers, respectively [17].

IP can be in different forms cash and non-cash [33]. Non-inclusion of the non-cash IP and the difference in the timeframe of reporting IP in the present study could explain the difference between this study and other findings.

There was a statistically significant pro-rich inequality in IP in Iran (high-income households compared to other households reported more IP) that is in line with another study in Iran [12] However, Szende et al. (2006), and Kankeu et al. (2016) ( found a pro-poor inequality in IP [11, 34]. In the study of Kankeu et al. (2016), many African countries experienced pro-poor inequality in IP [11]. Higher payment capacity of the privileged households, better and more access to health care providers, and more demand for high-quality services [35] could explain the positive NC for IP in the present study. In addition, differences in demanded services by high-income households compared to low-income households that could lead to a difference in IP [34, 36] is another explanation.

Inequality in IP in different provinces showed heterogeneous findings, in some provinces IP was more concentrated among high-income households (positive NC), in other provinces low-income households reported more IP (negative NC) and the prevalence of IPs in the rest of the provinces was same across different income groups. Differences in cultural [28, 36, 37] and economic characteristics of provinces that could shape the demand and sensitivity of individuals for IP could explain this heterogeneous inequality pattern across the country. Interestingly, while urban areas revealed a pro-rich inequality in IP, rural areas showed a concentration of IP in low-income households however the later inequality in the rural areas was not statistically significant. It seems in rural areas, factors such as the implementation of a family physician plan [38], referral systems and utilization from government health care providers lead to an equal exposure of rural residents from various economic classes to IP. Since IP is related to the demand for high-quality services [39] it seems in urban areas, due to the higher payment capacity of high income households and better access to healthcare providers, these households seek for high-quality services and reported more IP. In addition, not waiting in the waiting list through the referral system and seeking demands out of the referral system that could be more likely to bear IP is another possibility of pro-rich IP inequality in urban areas.

Inequality decomposition showed that household income was the most driving factor in the pro-rich inequality. Having higher income was related to the increased inequality in IP. However, analysis by year showed that, in 2017, the share of income in explaining observed inequality was lower compared to other two years; income was still the main contributor in the observed inequality. Also, Kankeu et al. found that the economic status (the poverty index) was the main contributor to explaining the pro-poor observed inequality in many African countries [11]. Limitations in access to health care in the low-income groups, and consequently, having unmet needs could lead to less use of care and fewer reporting of IP in these groups. Supply-side motivation and requests for more IP by providers from high-income households due to their payment ability could be the next explanation. As Kankeu et al. found, supply-side factors are among of the main contributors to the pro-poor inequality in many African countries [11]. Seeking high-quality services out of the formal government system and utilization from specific provider services, especially in the private sector, which could lead to more IP [26] is another possible reason. As Doshmangir et al. (2020) showed people who used private sector services had the highest IP [14]. Our findings emphasize the importance of payment ability in reporting IP. The only reason for seeking care outside the government system and paying IP is not patient motivation. In other words, although low-income individuals due to utilization from the public sector and referral system had a lower risk for IP [38] but even when they are not satisfied with health services quality in the public sector, limited payment capacity would hinder them from seeking their needs outside of defined public system [40] and consequently, reporting a lower IP.

Male-headed households increased the inequality in IP. Other studies showed that gender is related to IP [3, 28, 31, 41]. Having a higher payment capacity due to a higher income in male-headed households might explain this finding. Differences in the health services needed by the households whose heads were male compared to households whose heads were female [14, 30, 34] could be another reason for our finding. However, the yearly analysis showed that the share of sex in the explaining of IP inequality decreased. It could be explained by reduction of poor female during 2016 to 2018. The female head of household compared to male head of households were more disadvantaged economically, but during these years this situation has been relieved.

Although the probability of IP in 2018 and 2017 and the total years of the study, led to a reduction in IP prevalence. Differences in the economic status of the surveyed households in different years can be considered as possible reasons for the changes in IP prevalence in these years.

Although in different years education of the household head showed different contributions on the IP inequality (more effect in 2018), totally it was related to the reduction in the pro-rich IP inequality. As noted above, more payment capacity could explain more IP in educated household heads compared to illiterate ones. In addition, educated household heads without an academic degree compared to educated households head with an academic degree led to an increase in IP inequality that could be due to differences in the used health care services.

There were remarkable changes in the share of province in IP inequality. While in 2016, this variable had a small contribution to the IP's inequality, in 2017 and 2018 its share increased substantially. This study conducted in three different years after Health Transformation Plan (HTP). IP prevalence has shown heterogeneous pattern in other studies' results. In a national study one year after HTP (2014) a decreased pattern in IP was reported [42], another study in Kurdistan province in 2017, revealed that there was no IP [32, 43], however, another national study in 2018, showed that 22.8% of respondents have been exposed to IP [32]. Differences among provinces in terms of economic factors could be related to the change of provinces' contribution to IP inequality between 2016 and 2017. In other words, it seems increasing formal tariffs especially for inpatient services in HTP [32, 44] was not enough to stop providers from requesting IP. As other studies have shown providers inclination for IP is related to economic status of patients. So, IP request based on the differences in the economic position of provinces could explain this jump in province contribution between 2016 and 2017. Another reason could be seek in differences in the type of utilized healthcare services in these years. As Meskarpour et al. revealed IP is more prevalent in inpatient services compared to outpatient health care services [42].

Analysis based on the three years pooled data showed a heterogenous pattern of IP inequality among provinces. Our results also showed that province of residence had a positive contribution to the IP inequality. In line with our findings, the study of Kankeu et al. (2016) revealed that regional disparity was a significant driving factor for their pro-poor observed inequality in IP and there was an inequality heterogeneity among various African countries [11]. Differences in supply-side variables including providers' motivations [39] as well as demand-side variables in these provinces could be a possible reason for the present findings. Yang et al. (2013) showed physicians stated that IP is related to the environmental quality of which the services are provided and it is not related to providing care by them [45]. Place of residence could be related to IP in various ways. Differences in the needs and expectations of individuals and especially cultural differences in the provinces could explain differences in IP inequality [18, 41].

Having a family member over 65 years old leads to a decrease in IP inequality (totally and every year of the study). Among the possible reasons for the present finding may be a lower income level of households with a head over 65 years old.

Although, the pooled results showed a negligible share for urban residency on the IP inequality, the yearly analysis revealed its positive contribution on the measured inequality in 2016 and 2017 and its negative contribution in 2018. The reason behind this could be attributed to the economic position of urban residents compared to rural ones. As the appendix results show, compared to 2016 and 2017, in 2018, most of economically disadvantaged individuals lived in the urban areas. The diminishing concentration of the community wealth among urban residents during these years has been documented by other studies in Iran [46, 47]. Based on Fallahati et al., economic shocks in terms of increased exchange rate (two shocks recently in 2017) have affected negatively Iranian residents and it was seen that the urban residents were influenced more compared to rural areas. The more increased Gini Index in the urban areas compared to the rural districts could show this effect [46].

Although the most important strengths of the present study were using a large national pooled data set and the decomposition of IP inequality, some limitations should be considered. First, non-cash payments were not measured in the present study, while these payments are also part of IP. Second, in the present study, some people, especially those in low-income groups, may not have been able to use the services due to the provider's request for IP, and as a result have not made IP, which can lead to underestimating IP in Iran. Therefore, it is necessary to examine providers' requests for IP in the future studies to show a better picture of the extent of this problem in the Iranian health system. Finally, some other factors such as culture, attitudes of individuals, etc. are supposed to affect IP that we could not include in the current study [32].

## Conclusion

The results of the present study showed that there is a high prevalence and significant inequality in IP in the Iranian health system. On the whole, inequality was pro-rich. This may lead to increasing inequality in access to quality services in the country. Our findings showed that previous health policies such as regulatory tools, and the health transformation plan (HTP) have not been able to control IP in the health sector in the desired way. It seems that consumer-side policies focusing on affluent households, and high-risk provinces can play an important role in controlling this phenomenon.

## Supplementary Information

**Additional file 1.**

## Data Availability

The data that support the findings of this study is public and is available from ISC. Also, data are available from the corresponding author upon reasonable request.

## Abbreviations

IP: Informal payments
HIES: Health Income and Expenditures Survey
ISC: Iran Statistical Center
OOP: Out-of-pocket payments
HTP: Health Transformation Plan
NC: Normalized Concentration Index
RC: Reference Category
ME: Marginal Effect
CI: Concentration Index
AC: Absolute Contribution
SPC: Summed Percentage Contribution
