# Supplementary material for: Socioeconomic inequality in informal payments for health services among Iranian households: a national pooled study
Source: BMC Public Health. 2023 Feb 23;23:381. doi: 10.1186/s12889-023-15071-6 (PMC9948334; doi:10.1186/s12889-023-15071-6)
Supplement: Supplementary file 1 — Additional file 1. [file 12889_2023_15071_MOESM1_ESM.doc]

**Appendix1: Decomposition analysis of socioeconomic inequality in informal payments among Iranian households by years (2016-18)**

|  | **2016** | | | | | **2017** | | | | | **2018** | | | | | |
| --- | --- | --- | --- | --- | --- | --- | --- | --- | --- | --- | --- | --- | --- | --- | --- | --- |
| **Variables** | E | CI (Ck) | AC | PC | **SPC** | E | CI (Ck) | AC | PC | **SPC** | | E | CI (Ck) | AC | PC | **SPC** |
| **Sex (RC: Female)** |  |  |  |  |  |  |  |  |  |  | |  |  |  |  |  |
| Male | 0.27 | 0.35 | 0.09 | 53.15 | **53.15** | 0.13 | 0.34 | 0.05 | 28.13 | **28.13** | | 0.08 | 0.32 | 0.03 | 15.76 | **15.76** |
| **Age of Head of Household (RC: >65 years)** |  |  |  |  |  |  |  |  |  |  | |  |  |  |  |  |
| ≤35 | 0.05 | -0.13 | -0.01 | 3.86 | **11.74** | 0.00 | -0.12 | 0.00 | 0.21 | **-0.90** | | 0.05 | -0.16 | -0.01 | -4.62 | **-1.75** |
| 36-50 | 0.05 | 0.13 | 0.01 | 3.79 |  | -0.04 | 0.09 | 0.00 | -2.15 |  |  | 0.05 | 0.12 | 0.01 | 3.66 |  |
| 51-65 | 0.06 | 0.12 | 0.01 | 4.08 |  | 0.01 | 0.12 | 0.00 | 1.05 |  |  | -0.01 | 0.12 | 0.00 | -0.79 |  |
| **Education of head of household (RC: Academic)** |  |  |  |  |  |  |  |  |  |  | |  |  |  |  |  |
| Illiterate | 0.08 | -0.49 | -0.04 | -20.75 | **-18.66** | 0.06 | -0.51 | -0.03 | -19.81 | **-16.24** | | 0.10 | -0.51 | -0.05 | -30.46 | **-30.84** |
| Non academic | 0.04 | 0.08 | 0.00 | 2.09 |  | 0.13 | 0.04 | 0.01 | 3.57 |  |  | 0.18 | 0.00 | 0.00 | -0.38 |  |
| **Employment of head of household (RC: Unemployed)** |  |  |  |  |  |  |  |  |  |  | |  |  |  |  |  |
| Employed | -0.16 | 0.11 | -0.02 | -10.04 | **-10.04** | 0.05 | 0.10 | 0.01 | 3.18 | **3.18** | | -0.09 | 0.13 | -0.01 | -7.25 | **-7.25** |
| **Place of residence (RC: Rural)** |  |  |  |  |  |  |  |  |  |  | |  |  |  |  |  |
| Urban | 0.09 | 0.46 | 0.04 | 23.71 | **23.71** | 0.03 | 0.45 | 0.01 | 8.38 | **8.38** | | 0.12 | -0.48 | -0.06 | -33.87 | **-33.87** |
| **Income quintiles (RC: 5th)** |  |  |  |  |  |  |  |  |  |  | |  |  |  |  |  |
| 1st | -0.13 | -0.94 | 0.13 | 70.81 | **120.81** | -0.11 | -0.94 | 0.10 | 62.99 | **95.42** | | -0.20 | -0.20 | 0.18 | 111.47 | **160.00** |
| 2nd | -0.11 | -0.66 | 0.08 | 42.45 |  | -0.06 | -0.66 | 0.04 | 22.49 |  |  | -0.15 | -0.15 | 0.09 | 52.00 |  |
| 3rd | -0.10 | -0.27 | 0.03 | 14.98 |  | -0.08 | -0.27 | 0.02 | 13.14 |  |  | -0.11 | -0.16 | 0.02 | 10.53 |  |
| 4th | -0.06 | 0.21 | -0.01 | -7.42 |  | -0.02 | 0.21 | -0.01 | -3.19 |  |  | -0.07 | 0.31 | -0.02 | -14.00 |  |
| **Having an < 12 years old member in household (RC: No)** |  |  |  |  |  |  |  |  |  |  | |  |  |  |  |  |
| Yes | 0.08 | 0.03 | 0.00 | 1.17 | **1.17** | 0.05 | 0.00 | 0.00 | 0.02 | **0.02** | | -0.04 | 0.02 | 0.00 | -0.37 | **-0.37** |
| **Having an >65 years old member in household (RC: No)** |  |  |  |  |  |  |  |  |  |  | |  |  |  |  |  |
| Yes | 0.10 | -0.18 | -0.02 | -10.39 | **-10.39** | 0.10 | -0.16 | -0.02 | -9.64 | **-9.64** | | 0.07 | -0.17 | -0.01 | -6.81 | **-6.81** |
| **Having a member with special disease in household (RC: No)** |  |  |  |  |  |  |  |  |  |  | |  |  |  |  |  |
| Yes | 0.01 | 0.19 | 0.00 | 1.24 | **1.24** | 0.01 | 0.18 | 0.00 | 1.15 | **1.15** | | 0.01 | 0.25 | 0.00 | 1.03 | **1.03** |
| **Health insurance coverage (RC: No)** |  |  |  |  |  |  |  |  |  |  | |  |  |  |  |  |
| Yes | -0.01 | -0.05 | 0.00 | 0.27 | **0.27** | 0.07 | 0.00 | 0.00 | -0.12 | **-0.12** | | -0.02 | 0.09 | 0.00 | -1.19 | **-1.19** |

**E: Elasticity; Abbreviations: RC: Reference Category, CI: Concentration Index (C_k_), AC: Absolute contribution, SPC: Summed percentage contribution**

**Appendix1: Decomposition analysis of socioeconomic inequality in informal payments among Iranian households by years (2016-18) (continue…)**

| **Province (RC: Alborz)** |  |  |  |  |  |  |  |  |  |  |  |  |  |  |  |
| --- | --- | --- | --- | --- | --- | --- | --- | --- | --- | --- | --- | --- | --- | --- | --- |
| Markazi | 0.01 | -0.11 | 0.00 | -0.32 | **1.50** | 0.00 | -0.20 | 0.00 | -0.27 | **33.37** | 0.01 | -0.09 | 0.00 | -0.52 | **38.46** |
| Guilan | -0.06 | -0.10 | 0.01 | 3.30 |  | 0.00 | -0.11 | 0.00 | 0.00 |  | -0.04 | -0.15 | 0.01 | 4.00 |  |
| Mazandaran | -0.04 | 0.07 | 0.00 | -1.64 |  | -0.08 | 0.13 | -0.01 | -6.03 |  | -0.01 | 0.10 | 0.00 | -0.86 |  |
| Azerbaijan, East | -0.03 | -0.05 | 0.00 | 0.70 |  | -0.04 | -0.13 | 0.00 | 3.08 |  | -0.03 | -0.12 | 0.00 | 1.96 |  |
| Azerbaijan, West | 0.02 | -0.33 | -0.01 | -3.66 |  | 0.03 | -0.22 | -0.01 | -3.69 |  | 0.01 | -0.28 | 0.00 | -1.20 |  |
| Kermanshah | 0.01 | -0.06 | 0.00 | -0.49 |  | 0.02 | -0.14 | 0.00 | -1.48 |  | -0.01 | 0.01 | 0.00 | -0.02 |  |
| Khuzestan | -0.06 | 0.04 | 0.00 | -1.28 |  | 0.00 | 0.01 | 0.00 | 0.00 |  | -0.07 | 0.03 | 0.00 | -1.20 |  |
| Fars | 0.02 | 0.05 | 0.00 | 0.60 |  | 0.00 | 0.03 | 0.00 | -0.08 |  | 0.00 | 0.01 | 0.00 | 0.02 |  |
| Kerman | 0.00 | -0.47 | 0.00 | -1.29 |  | -0.01 | -0.50 | 0.01 | 4.05 |  | -0.03 | -0.46 | 0.01 | 8.91 |  |
| Khorasan, Razavi | -0.05 | -0.16 | 0.01 | 4.51 |  | 0.02 | -0.10 | 0.00 | -1.20 |  | 0.01 | -0.07 | 0.00 | -0.57 |  |
| Isfahan | 0.04 | 0.07 | 0.00 | 1.51 |  | 0.00 | 0.06 | 0.00 | -0.15 |  | -0.03 | 0.06 | 0.00 | -1.11 |  |
| Sistan and Baluchestan | -0.05 | -0.34 | 0.02 | 8.72 |  | -0.02 | -0.15 | 0.00 | 1.49 |  | -0.06 | -0.54 | 0.03 | 20.05 |  |
| Kurdistan | 0.01 | -0.15 | 0.00 | -0.89 |  | 0.01 | -0.11 | 0.00 | -0.36 |  | 0.00 | -0.12 | 0.00 | -0.13 |  |
| Hamadan | 0.02 | -0.12 | 0.00 | -1.35 |  | -0.01 | -0.05 | 0.00 | 0.32 |  | 0.00 | -0.21 | 0.00 | -0.17 |  |
| Chahar Mahaal and Bakhtiari | 0.01 | 0.06 | 0.00 | 0.22 |  | 0.01 | 0.05 | 0.00 | 0.21 |  | 0.01 | -0.02 | 0.00 | -0.13 |  |
| Lorestan | 0.00 | -0.16 | 0.00 | -0.27 |  | 0.01 | -0.21 | 0.00 | -0.95 |  | -0.01 | -0.21 | 0.00 | 1.67 |  |
| Ilam | 0.00 | -0.22 | 0.00 | 0.51 |  | 0.00 | -0.22 | 0.00 | -0.02 |  | 0.00 | -0.20 | 0.00 | 0.58 |  |
| Kohgiluyeh and Boyerahmad | 0.01 | -0.16 | 0.00 | -0.48 |  | 0.01 | -0.13 | 0.00 | -0.47 |  | 0.00 | -0.03 | 0.00 | -0.06 |  |
| Bushehr | 0.00 | 0.03 | 0.00 | 0.01 |  | 0.01 | 0.05 | 0.00 | 0.17 |  | 0.00 | 0.01 | 0.00 | 0.01 |  |
| Zanjan | -0.01 | -0.19 | 0.00 | 1.10 |  | -0.04 | -0.14 | 0.01 | 3.25 |  | -0.01 | -0.06 | 0.00 | 0.55 |  |
| Semnan | 0.00 | -0.32 | 0.00 | 0.32 |  | 0.00 | -0.29 | 0.00 | 0.19 |  | 0.00 | -0.25 | 0.00 | -0.40 |  |
| Yazd | -0.01 | -0.02 | 0.00 | 0.17 |  | -0.03 | -0.01 | 0.00 | 0.21 |  | -0.02 | 0.04 | 0.00 | -0.42 |  |
| Hormozgan | 0.01 | -0.15 | 0.00 | -0.92 |  | -0.01 | -0.05 | 0.00 | 0.24 |  | -0.01 | 0.00 | 0.00 | -0.01 |  |
| Tehran | -0.01 | 0.48 | -0.01 | -4.03 |  | 0.10 | 0.47 | 0.05 | 28.13 |  | -0.01 | 0.47 | 0.00 | -2.23 |  |
| Ardabil | 0.01 | -0.22 | 0.00 | -1.09 |  | 0.02 | -0.17 | 0.00 | -1.98 |  | 0.02 | -0.08 | 0.00 | -0.88 |  |
| Qom | 0.01 | 0.01 | 0.00 | 0.02 |  | -0.05 | -0.01 | 0.00 | 0.43 |  | -0.03 | -0.01 | 0.00 | 0.24 |  |
| Qazvin | -0.02 | -0.06 | 0.00 | 0.61 |  | -0.07 | -0.10 | 0.01 | 4.39 |  | -0.01 | 0.00 | 0.00 | -0.02 |  |
| Golestan | 0.01 | -0.32 | 0.00 | -2.42 |  | -0.01 | -0.25 | 0.00 | 2.04 |  | 0.00 | -0.18 | 0.00 | -0.03 |  |
| Khorasan, North | 0.00 | -0.23 | 0.00 | -0.63 |  | 0.01 | -0.23 | 0.00 | -1.28 |  | -0.01 | -0.25 | 0.00 | 1.60 |  |
| Khorasan, South | 0.00 | -0.44 | 0.00 | -0.04 |  | -0.01 | -0.44 | 0.01 | 3.14 |  | -0.03 | -0.42 | 0.01 | 8.84 |  |
| Explained |  |  | 0.29 |  | 174.50 |  |  | **0.23** |  | 142.75 |  |  | 0.22 |  | 133.17 |
| Residual |  |  | -0.12 |  | -74.5 |  |  | **-0.07** |  | -42.75 |  |  | -0.05 |  | -33.17 |
| Total |  |  | 0.18 |  | 100 |  |  | **0.16** |  | 100 |  |  | 0.17 |  | 100.00 |
